# Genetic Diagnosis of Familial Hypercholesterolemia in Asia

**DOI:** 10.3389/fgene.2020.00833

**Published:** 2020-07-24

**Authors:** Chin-Chou Huang, Min-Ji Charng

**Affiliations:** ^1^Division of Cardiology, Department of Medicine, Taipei Veterans General Hospital, Taipei, Taiwan; ^2^Faculty of Medicine, School of Medicine, National Yang-Ming University, Taipei, Taiwan; ^3^Cardiovascular Research Center, National Yang-Ming University, Taipei, Taiwan; ^4^Institute of Pharmacology, National Yang-Ming University, Taipei, Taiwan

**Keywords:** apolipoprotein B, Asia, diagnosis, familial hypercholesterolemia, gene, low-density lipoprotein receptor, proprotein convertase subtilisin/kexin type 9, mutation

## Abstract

Familial hypercholesterolemia (FH) is a common genetic disease with an incidence of about 1 in 200–500 individuals. Genetic mutations markedly elevate low-density lipoprotein cholesterol and atherosclerotic cardiovascular disease (ASCVD) in FH patients. With advances in clinical diagnosis and genetic testing, more genetic mutations have been detected, including those in low-density lipoprotein receptor (*LDLR*), apolipoprotein B (*APOB*), proprotein convertase subtilisin/kexin type 9 (*PCSK9*), and so on. Globally, most FH patients remain undiagnosed, untreated, or inappropriately treated. Recently, there was a Global Call to Action by the Global Familial Hypercholesterolemia Community to reduce the health burden of FH. Asia, despite being the most populous continent with half of the global population, has low FH detection rates compared to Western countries. Therefore, we aimed to review the current status of FH genetic diagnosis in Asia to understand the gaps in FH diagnosis and management in this region.

## Introduction

Familial hypercholesterolemia (FH) is a common genetic disease with an incidence of about 1 in 200–500 individuals (Marks et al., 2003; Benn et al., 2012). Lipid metabolism changes in FH patients, including reduced low-density lipoprotein receptor (LDLR)-mediated low-density lipoprotein cholesterol (LDL-C) catabolism, impaired apolipoprotein B (APOB)-mediated LDL clearance, and increased proprotein convertase subtilisin/kexin type 9 (PCSK9) levels, mediate post-translational destruction of LDLRs (Chiou and Charng, 2016, Sturm et al., 2018). Genetic mutations in FH patients have been detected in *LDLR*, *APOB*, and *PCSK9*, and are either in a heterozygous (HeFH) or homozygous (HoFH) FH form (Chiou and Charng, 2016; Sturm et al., 2018, Di Taranto et al., 2020; Nawawi et al., 2020). These genetic mutations result in a marked elevation of LDL-C levels and atherosclerotic cardiovascular disease (ASCVD) if these patients are left underdiagnosed and undertreated (Nordestgaard et al., 2013; Gidding et al., 2015, Elkins and Fruh, 2019). For patients harboring FH-causing variants, the risk of ASCVD may be increased by 4.4- to 6.8- fold (Lee et al., 2019). Therefore, early diagnosis of FH is important.

However, globally, the majority of FH patients remain undiagnosed, untreated, or insufficiently treated (Nordestgaard et al., 2013; Khera et al., 2016, Cao et al., 2018; Wang et al., 2019). Based on clinical criteria, FH is not rare in the Asian population (Wang et al., 2019). However, around 42.5% to 62.5% of the FH patients remain underdiagnosed, according to definitions by the Dutch Lipid Clinic Network (DLCN) and Simon Broome (SB). This finding supports the importance of genetic testing for the diagnosis of FH (Cao et al., 2018).

Furthermore, there are great advances in diagnostic technologies, such as next generation sequencing (NGS) (Di Resta and Ferrari, 2018), to improve the genetic diagnosis of FH. In the era of targeted and personalized medicine (Prodan Žitnik et al., 2018), it will be useful to identify different patient types to aid the selection of safer and more effective treatments.

Recently, there was a Global Call to Action by the Global Familial Hypercholesterolemia Community to reduce the health burden of FH (Wilemon et al., 2020). Asia is the most populous continent in the world, with more than half of the world’s population. It is therefore important to better understand the current status of FH diagnosis and management in Asia. Here, we aimed to review the current status of FH genetic diagnosis in Asia.

## FH Detection in Asia

There are two related studies on FH detection in Asia (Nordestgaard et al., 2013; Pang et al., 2019). Nordestgaard et al. (2013) reported the estimated number and percentage of individuals diagnosed with FH globally. Among roughly 200 countries/territories worldwide, data were only available in 22 countries/territories, including 3 in Asia. The data from Asian countries were derived from the second WHO consultation on FH in 1998. The estimated percentage of individuals diagnosed with FH was only 1% (*n* = 14,100) in Hong Kong, <1% (*n* = 46,300) in Taiwan, and <1% (*n* = 254,800) in Japan (Nordestgaard et al., 2013).

Another study in 2019 compared FH health care in 12 countries, including 9 countries selected in the Asia-Pacific region, and 2 in the Southern Hemisphere and United Kingdom (UK). In the United Kingdom, which was the benchmark, the estimated percentage of individuals diagnosed with FH was 10–20%. In non-Asian countries, the estimated percentage was 5.3% in South Africa, 4% in Australia, 2.9% in Brazil, and 2.5% in New Zealand. In Asian countries, however, the estimated percentage was only 3.8% in Taiwan, 2.2% in Hong Kong, 1.4% in Malaysia, 1% in Japan, <1% in China, <1% in the Philippines, and <1% in Vietnam. Although there is a continuous improvement in FH detection rates, compared to the 2013 data, there are still important gaps in comparison to other non-Asian countries and the United Kingdom (Pang et al., 2019).

## Awareness and Knowledge of FH in Asia

The awareness and knowledge about FH have been surveyed among physicians across Asia (Pang et al., 2015, 2017). One anonymous internet-based survey involving 230 physicians was reported in 2015 (Pang et al., 2015). These physicians were selected from Japan, South Korea, and Taiwan. Only a small proportion of them was aware of the heritability (47%), prevalence (27%), risk of FH-related cardiovascular disease (13%), and FH specialists in their geographic area (35%) (Pang et al., 2015).

Another survey was done with 1,078 physicians and reported in 2017 (Pang et al., 2017). These physicians were from 10 different countries/regions, including Australia, Japan, Malaysia, South Korea, Philippines, Hong Kong, China, Vietnam, Taiwan, and the United Kingdom. Self-perceived FH familiarity was only 34% and there were no significant differences among most countries (except in Japan and China, where the value was lower) and the United Kingdom. Physicians from the Asia-Pacific region were significantly worse at selecting the correct FH descriptions (72%), compared to those from the United Kingdom (89%) (*P* = 0.001). FH guideline awareness was significantly lower in physicians from the Asia-Pacific region (35%), compared to those from the United Kingdom (61%) (*P* < 0.001). The awareness of lipid specialists for referral or medical advice was also significantly lower in physicians from the Asia-Pacific region (35%), compared to those from the United Kingdom (50%) (*P* = 0.003) (Pang et al., 2017).

These studies showed that the awareness, knowledge, and perception about FH were still low among Asian physicians, particularly in developing countries (Pang et al., 2015, 2017). More efforts are needed for improvement in these parameters, where extensive education and training programs could be of help.

## Genetic Diagnosis of FH in Asia

### Taiwan

FH genetic studies in Taiwan are summarized in Table 1.

**TABLE 1:** Genetic diagnosis of familial hypercholesterolemia in Taiwan.

**Case numbers**	**Age**	**Male**	**Diagnosis criteria**	**Genotyping**	**Main Findings**	**References**
51	57 (26–84)	22 (43.1%)	Simon Broome criteria	Sanger sequencing for *LDLR* and *APOB* mutations	Thirteen different functional mutations in *LDLR* and one mutation in *APOB* were found in 21 patients (41.2%).	Charng et al., 2006
87	42 (14–70)	46 (52.9%)	Clinical diagnosed autosomal dominant hypercholesterolemia	Sanger sequencing for *LDLR*, *APOB*, and *PCSK9* mutations	Genetic mutations were identified in 55 members (60%) from 18 families.	Yang et al., 2007
102	44 (34–54)	40 (39.2%)	Simon Broome criteria	Sanger sequencing and MLPA	Frequency of large *LDLR* rearrangement was approximately 8% in Taiwanese patients with FH.	Chiou and Charng, 2010
208	NM	NM	Simon Broome criteria	Sanger sequencing and MLPA	*LDLR* mutations were found in 118 probands (56.7%), consisting of 61 different loci, and *APOB* c.10579 C > T (p.R3527W) mutations in 12 probands (5.8%).	Chiou and Charng, 2012
35 (first) and 125 (second)	42 (second)	46 (36.8%) (second)	Simon Broome criteria	A custom DNA resequencing array to detect mutations on 3 FH-causing genes (*LDLR*, *APOB*, and *PCSK9*) and 290 known insertion/deletion mutations on *LDLR*	The average microarray call rate was 98.45% and the agreement between microarray and capillary sequencing was 99.99%.	Chiou et al., 2011
120 (first) and 185 (second)	54 (second)	77 (41.6%) (second)	Dutch Lipid Clinic Network criteria	A custom Agena iPLEX assay to detect 68 point mutations on FH-causing genes	The detection sensitivity and specificity rates of Agena iPLEX were 92.5% and 100%, respectively, in the blind study.	Chiou and Charng, 2017
13 (first) and 28 (second)	NM	NM	Dutch Lipid Clinic Network score ≥ 5	NGS for *LDLR*, *APOB* and *PCSK9* genes	NGS correctly identify all the variants, including big duplications and deletions.	Hsiung et al., 2018

*APOB, apolipoprotein B; DNA, deoxyribonucleic acid; LDL-C, low-density lipoprotein cholesterol; *LDLR*, low-density lipoprotein receptor; MLPA, Multiplex ligation dependent probe analysis; NGS, next generation sequencing; NM, not mention; *PCSK9*, proprotein convertase subtilisin/kexin type 9; SNP, single nucleotide polymorphism.*

There are some studies that investigated the genetic mutations of FH patients and the development of genetic diagnostic methods in Taiwan (Charng et al., 2006; Yang et al., 2007, Chiou and Charng, 2010; Chiou et al., 2011, Chiou and Charng, 2012, 2017, Hsiung et al., 2018).

In a small population of 51 unrelated FH patients in Taiwan, *LDLR* and *APOB* mutations were screened using Sanger sequencing. Among these patients, 13 different functional mutations in *LDLR* and 1 in *APOB* were found in 21 patients (41.2%). For the *LDLR* mutations, there were 10 single-point missense mutations, 1 two-point mutation in the same allele, 1 non-sense mutation, and 1 frame-shift mutation. The study discovered 3 novel mutations, including 2 missense mutations [*LDLR* c.1592 T > A (p.M531K) and *LDLR* c.1597 T > C (p.W533R)] and 1 frame-shift mutation [*LDLR* c.1953,1954 del [TA] (p.M652Fs)] (Charng et al., 2006).

In another study, 87 FH patients from 30 unrelated Taiwanese families were screened for *LDLR*, *APOB*, and *PCSK9* mutations via Sanger sequencing. Genetic mutations were identified in 55 members (60%), including 6 previously reported *LDLR* mutations [*LDLR* c.383 G > A (p.C128Y), *LDLR* c.268 G > A (p.D90N), *LDLR* c.1216 C > T (p.R406W), *LDLR* c.1448 G > A (p.W483X), *LDLR* c.571 C > T (p.G191X), and *LDLR* c.1285 G > A (p.V429L)], 2 novel *LDLR* mutations [*LDLR* c.1954 del AT (p.M631Fs) and *LDLR* c.1586 + 5 G > C], 1 known [*APOB* c.10579 C > T (p.R3527W)], and 1 novel missense mutations [*APOB* c.10718 C > T (p.R3567W)] in the *APOB* gene. The study found no *PCSK9* gene mutations in this Taiwanese patient cohort (Yang et al., 2007).

One hundred two unrelated probands, who fulfilled the FH diagnostic criteria, were tested for mutations using Sanger sequence analysis and multiple ligation-dependent probe amplification (MLPA). Gene mutations were detected in 60 probands (58.8%), including *LDLR* mutations in 52 probands (51.0%) and the *APOB* c.10579 C > T (p.R3527W) mutation in 8 probands (7.8%). Among the 42 probands with mutations undetected by exome sequence analysis, 8 had abnormal MLPA patterns, including 2 with exon 6 to 18 deletions, 2 with exon 9 deletions, 1 with exon 6 to 8 deletions, 1 with exon 11 deletions, 1 with exon 3 to 5 duplications, and 1 with exon 7 to 12 duplications. This study found that the frequency of large *LDLR* rearrangements was approximately 8% in Taiwanese FH patients (Chiou and Charng, 2010).

In 208 Taiwanese patients with clinically diagnosed FH, common FH mutations were investigated using Sanger sequencing and MLPA. The top three most common mutations were *LDLR* c.986G > A (p.C329Y) (13.1%), c.1747C > T (p.H583Y) (10.8%), and *APOB* c.10579C > T (p.R3527W) (9.2%). Among these patients, 118 probands (56.7%) had *LDLR* mutations and 12 (5.8%) had the *APOB* c.10579 C > T (p.R3527W) mutation. Three novel mutations were discovered, including *LDLR* c.64 del G (p.A22Fs), *LDLR* c.1661 C > T (p.S554L), and *LDLR* c.2099 A > G (p.D700G). One haplotype [CAAGCCCCATGG/(dTA)n-112nt] was associated with *LDLR* c.1747 C > T (p.H583Y), and two were associated with *LDLR* c.986 G > A (p.C329Y). In comparison to southern Chinese patients, the same *LDLR* binding-domain pattern was associated with *APOB* c.10579 C > T (p.R3527W) in Taiwanese patients. The study found different common FH mutation-associated haplotypes and haplotype homologies in Taiwan and southern China, suggesting multiple historical migrations of Taiwanese and the presence of common ancestors in southern China (Chiou and Charng, 2012).

Some studies have investigated genetic diagnosis techniques in Taiwan. A custom DNA resequencing array was designed to detect mutations on all three FH-causing genes (*LDLR*, *APOB*, and *PCSK9*) and 290 known *LDLR* insertion/deletion mutations in the Taiwanese patients. In previously sequenced patients, the average call rate was 98.45% and the agreement between microarray and capillary sequencing was 99.99%. In screening patients blindly, the FH array detected at least 1 mutation in 77.5% of patients clinically diagnosed with definite FH and in 52.9% of patients with probable FH. The high-throughput FH resequencing array detected *LDLR*, *APOB*, and *PCSK9* with high efficiency and accuracy and identifies disease-causing mutations (Chiou et al., 2011).

A mass spectrometric assay (Agena iPLEX assay) was designed to simultaneously diagnose 68 point mutations in FH-causing genes in Taiwanese patients. In the first part of the study, only 1 discrepancy was found between the mass spectrometry and Sanger sequencing data of 180 previously sequenced patients. In the second part, 62 probands with mutations were identified by both techniques out of 185 FH probands, with only 5 mutations detected by Sanger sequencing. The sensitivity and specificity of mass spectrometry were 92.5% and 100%, respectively. This study found great potential for this assay due to its low cost, speed (around 1 day), and flexibility for FH genetic screening in Taiwanese patients (Chiou and Charng, 2017).

Probes for NGS were designed to capture the whole *LDLR* gene and all coding sequences of *APOB* and *PCSK9* in Taiwanese patients. These probes correctly identified all the variants in 13 DNA samples, including 3 large duplications and 2 large deletions. In a new cohort of 28 unrelated FH patients with a Dutch Lipid Clinic Network score ≥ 5, they identified the causative variants in 21 unrelated probands. Five of them carried the novel splice site variant c.1186 + 2 T > G in *LDLR*. This study showed that this panel can comprehensively detect disease-causing variants in *LDLR*, *APOB*, and *PCSK9* in FH patients (Hsiung et al., 2018).

With the advances in molecular diagnostic methods, the detection rate of genetic mutations in clinically diagnosed FH has increased in Taiwan in recent years. However, there were still large proportions of FH patients that did not have genetic diagnoses. There were multiple *LDLR* and *APOB* mutations identified in Taiwan, including some novel mutations. However, *PCSK9* mutations, which had been reported in China and Japan, have not been identified in Taiwan.

### China

FH genetic studies in China are summarized in Table 2.

**TABLE 2:** Genetic diagnosis of familial hypercholesterolemia in China.

**Case numbers**	**Age**	**Male**	**Diagnosis criteria**	**Genotyping**	**Main Findings**	**References**
105	31.7	99 (94.3%)	Age ≤ 35 years and LDL-C ≥ 3.4 mmol/L	Genotyping for 9 genes, including *LDLR*, *APOB*, *PCSK9*, *APOE*, *STAP1*, *LIPA*, *LDLRAP1*, and *ABCG*5/8.	The prevalence of genetically confirmed FH was 38.1%.	Cao et al., 2018
285	49	173 (60.7%)	Dutch Lipid Clinic Network score ≥ 6	NGS for *LDLR*, *APOB* and *PCSK9* genes	The detection rate is 51.9% (148/285) with a total of 119 risk variants identified including 84 in *LDLR*, 31 in *APOB* and 4 in *PCSK9* gene.	Sun et al., 2018

*APOB, apolipoprotein B; FH, familial hypercholesterolemia; LDL-C, low-density lipoprotein cholesterol; *LDLR*, low-density lipoprotein receptor; *PCSK9*, proprotein convertase subtilisin/kexin type 9; NGS, next generation sequencing.*

In 105 young patients (age ≤ 35 years) with high LDL-C (LDL-C ≥ 3.4 mmol/L), genetic analysis was performed for 9 genes (*LDLR*, *APOB*, *PCSK9*, *APOE*, *STAP1*, *LIPA*, *LDLRAP1*, ABCG5 and ABCG8). Genetic mutations were detected in 40 patients (38.1%). This study found that the best LDL-C threshold was 4.56 mmol/L for genetically confirmed FH (Cao et al., 2018).

In a multi-center study in China, 285 unrelated index cases with clinical FH were analyzed by NGS for *LDLR*, *APOB*, and *PCSK9* mutations. Genetic mutations were detected in 148 cases (51.9%), including 84 (29.4%) in *LDLR*, 31 (10.9%) in *APOB*, and 4 (1.4%) in *PCSK9* genes. *LDLR* c.1448 G > A (p.W483X) was detected in 9 patients, which was the most frequent mutation. There were 8 novel mutations (7 *LDLR* and 1 *APOB*), which were considered as pathogenic by *in silico* analysis. There were 5 true *LDLR* homozygotes, 16 compound heterozygotes, and 13 double heterozygotes identified. The most severe phenotypes were noted in true *LDLR* homozygotes (Sun et al., 2018).

There was a review of 163 case reports about Chinese FH patients published before 2018. The mutation frequency was 82% in *LDLR*, 9% in *APOB*, and very rare in *PCSK9*. The study concluded that the epidemiological investigation of FH was not large-scale, FH recognition remained rudimentary, and guidelines for diagnosis and management of FH patients were incomplete in China (Peng et al., 2019).

In a previous systematic review of *LDLR* mutations in Chinese FH patients, a total of 74 studies were included. Among the 295 probands studied, 131 *LDLR* mutations were identified. Most of them were located in exon 4, and approximately 60% of them were missense mutations. There were 30 novel mutations and most of them were found pathogenic by *in silico* analysis. The major *LDLR* mutations were *LDLR* c.986 G > A (p.C329Y), *LDLR* c.1747 C > T (p.H583Y), and *LDLR* c.1879 G > A (p.A627T) (Jiang et al., 2015).

The majority of FH mutations found in Chinese were in *LDLR*, there was one in *APOB*, and were rare in *PCSK9*. The functionality of these novel mutations was predicted by *in silico* analysis. Functional testing or familial co-segregation analysis may be required to check the pathogenicity of these mutations.

### Hong Kong

FH genetic studies in the Hong Kong are summarized in Table 3.

**TABLE 3:** Genetic diagnosis of familial hypercholesterolemia in Hong Kong.

**Case numbers**	**Age**	**Male**	**Diagnosis criteria**	**Genotyping**	**Main Findings**	**References**
30	42 (11–80	17 (56.7%)	(1) Fasting plasma total cholesterol levels >7.5 mmol/L in adults (>6.5 mmol/L in individuals younger than 16 years) and having normal fasting plasma triglyceride levels, and (2) presence of tendon xanthomata in proband or first degree relative, or other family members having raised LDL-C inherited in a dominant pattern.	DNA sequencing for the promoter and 18 coding exons of the *LDLR* gene	*LDLR* mutations were diagnosed in 21 patients (70.0%). For the 9 clinically diagnosed FH patients with no detectable *LDLR* gene mutations, there was also no *APOB* R3500Q mutation detected.	Mak et al., 1998
94	51.9	42 (44.7%)	Dutch Lipid Clinic Network criteria	Sanger sequencing for *LDLR*, *APOB*, or *PCSK9* genes	Single mutation was detected in 54 probands (57.4%), including 51 in *LDLR* and 3 in *APOB*.	Tan et al., 2018
96	51.7	43 (44.8%)	Clinical diagnosis of FH or severe hypercholesterolemia	Family based cascade screening incorporating genetic testing	Forty-two distinct mutations were identified in 67% of the index FH cases. The majority of causative mutations were in *LDLR*.	Chan et al., 2019

*APOB, apolipoprotein B; DNA, deoxyribonucleic acid; LDL-C, low-density lipoprotein cholesterol; *LDLR*, low-density lipoprotein receptor; *PCSK9*, proprotein convertase subtilisin/kexin type 9; MLPA.*

Hu et al. reported the epidemiologic features of 252 HeFH patients from the Chinese population in Hong Kong. For patients aged ≥ 18 years, xanthomata was noted in 40.6% of males and 54.8% of females, while corneal arcus was noted in 81.2% of males and 66.9% of females. The incidence of coronary artery disease (CAD) was 9.9% and 8.5% in males and females, respectively. This study found that age and the presence of xanthelasmata were associated with the risk of CAD (Hu et al., 2013, 2016).

In one genetic study from Hong Kong, DNA sequencing was performed for the promoter and coding regions of the 18 exons of the *LDLR* gene in 30 Chinese FH patients. Overall, *LDLR* mutations were detected in 21 patients (70.0%). There were 18 mutations in the promoter and 10 exons. Besides, there were 6 polymorphisms with genotypic distributions similar to those in the Japanese population but different from those in the Western populations. For the 9 clinically diagnosed FH patients with no detectable *LDLR* gene mutations, there was also no *APOB* R3500Q mutations detected (Mak et al., 1998).

In another genetic study from Hong Kong, a total of 94 unrelated probands with clinical FH received genetic testing for *LDLR*, *APOB*, and *PCSK9* by Sanger sequencing. Single-gene mutations were detected in 54 probands (57.4%), including 51 (54.2%) in *LDLR* and 3 (3.2%) in *APOB*. Heterozygous *LDLR* mutations were the most common. Compound heterozygous mutations were detected in five cases. Double heterozygous mutations were detected in three cases. A homozygous mutation in exon 10 of *LDLR* [c.1474G > A (p.D492N)] was detected in one case (Tan et al., 2018).

In a recent study, Chinese subjects with clinical FH received family based cascade screening and genetic testing. Among 96 index cases, 42 distinct mutations were identified in 64 cases (67%). The majority of mutations were in *LDLR*, and the three most common mutations were *LDLR* c.1241 T > G (p.L414R), *LDLR* c.1474G > A (p.D492N), and *LDLR* c.682G > A (p.E228X). Nine novel *LDLR* mutations were identified. *APOB* c.10579 C > T (p.R3527W) was identified in 5% of the total cases (Chan et al., 2019).

### Japan

FH genetic studies in Japan are summarized in Table 4.

**TABLE 4:** Genetic diagnosis of familial hypercholesterolemia in Japan.

**Case numbers**	**Age**	**Male**	**Diagnosis criteria**	**Genotyping**	**Main Findings**	**References**
350	45	197 (56.3%)	Clinically diagnosed HeFH	Genetic analysis of *APOB* 3500 mutation	*APOB* 3500 mutation was not detected.	Nohara et al., 1995
200	45	90 (45.0%)	Clinically diagnosed HeFH	Denaturing gradient-gel electrophoresis, DNA sequencing and Southern blotting analysis	*LDLR* mutations were detected in 125 patients (62.5%). *APOB* mutation was not detected in this cohort.	Yu et al., 2002
25	32 (1–73)	12 (48.0%)	Clinically or genetically diagnosed HoFH	*LDLR* mutations were identified using the Invader assay method. Mutations in PCSK9 were detected by PCR- single-strand conformational polymorphism followed by direct sequence analysis.	There were 15 true homozygotes and 10 compound heterozygotes for *LDLR* mutations. Thirteen *LDLR* mutations were detected. Five of these patients had *PCSK9* mutation, including 2 patients with true homozygotes and 3 patients with compound heterozygotes. Three types of double heterozygotes for *LDLR* and *PCSK9* were detected.	Mabuchi et al., 2011
269	NM	NM	Clinically diagnosed HeFH	A direct sequence analysis for all 18 exons of *LDLR* and 12 exons of *PCSK9* gene	There were 11 *PCSK9* variants detected. *PCSK9* c.10 G > A (p.V4I) variant was linked to increased risk of coronary artery disease in patients aged ≥30 years and having *LDLR* mutations.	Ohta et al., 2016
801	NM	NM	The patients having at least two of the following factors: LDL-C ≥ 180 mg/dL, tendon/skin xanthomas, and familial history of FH or premature coronary artery disease within the second degree of kinship.	All coding regions and the exon-intron boundary sequence of the *LDLR* and *PCSK9* genes were examined.	Pathogenic variants in the *LDLR* and *PCSK9* genes were found in 296 (46%) and 51 (7.8%) of unrelated FH patients (*n* = 650), respectively.	Hori et al., 2019

*APOB, apolipoprotein B; DNA, deoxyribonucleic acid; FH, familial hypercholesterolemia; HeFH, heterozygous familial hypercholesterolemia; HoFH, homozygous familial hypercholesterolemia; LDL-C, low-density lipoprotein cholesterol; *LDLR*, low-density lipoprotein receptor; NM, not mention;PCSK9, proprotein convertase subtilisin/kexin type 9.*

In a previous report, 385 clinically diagnosed HeFH patients received genetic analysis for APOB 3500 mutations to estimate the frequency of familial defective apolipoprotein B-100 (FDB) in Japan. The study detected no APOB 3500 mutation in this cohort (Nohara et al., 1995).

In a study conducted in the Hokuriku district of Japan, 200 unrelated FH patients received genetic analysis for *LDLR* and *APOB* genes. *LDLR* mutations were detected in 125 patients (62.5%). There were 37 probable disease-causing mutations, 22 of which were novel. Common *LDLR* mutations included *LDLR* c. 2431 C > T (p.K811X) (19.5%), *LDLR* c. 2054 C > T (p.P685L) (6.0%), FH-Tonami-1 (6.0%), *LDLR* c.2141-3 C > A (5.5%), and FH-Tonami-2 (4.5%). *APOB* mutations were not detected in this cohort (Yu et al., 2002).

In another study conducted in the Hokuriku district of Japan, 25 clinical HoFH patients received genetic analysis reports. There were 15 true homozygotes and 10 compound heterozygotes for *LDLR* mutations. Five of these patients had *PCSK9* mutation [*PCSK9* c.94G > A (p.E32K)], including 2 true homozygotes and 3 compound heterozygotes (*LDLR* and *PCSK9*). *APOB* mutations were not detected in any FH patient. The study also confirmed a high incidence of HoFH (1/171,167) and HeFH (1/208) in the Hokuriku district of Japan (Mabuchi et al., 2011).

In 269 clinically diagnosed HeFH patients in Japan, genetic analyses were performed for *LDLR* and *PCSK9* gene mutations. Eleven PCSK9 variants were detected. The four most frequent *PCSK9* variants were c.63_64insCTG (p.L21_22insL), c.158 C > T (p.A53V), c.10 G > A (p.V4I), and c.94G > A (p.E32K). PCSK9 c.158 C > T (p.A53V) and c.63_64insCTG (p.L21_22insL) were in linkage disequilibrium with each other. Interestingly, the PCSK9 c.10 G > A (p.V4I) variant was linked to an increased risk of CAD in patients aged ≥ 30 years with *LDLR* mutations. The study suggested that *PCSK9* c.10 G > A (p.V4I) might affect *LDLR* metabolism and modify *LDLR* mutation phenotypes (Ohta et al., 2016).

Recently, 801 clinically diagnosed Japanese HeFH patients were analyzed for *LDLR* and *PCSK9* mutations. Among the 650 unrelated FH patients, *LDLR* pathogenic variants were identified in 296 patients (46%) and *PCSK9* pathogenic variants were identified in 51 patients (7.8%). *PCSK9* c.94G > A (p.E32K) was the most frequently detected pathogenic *PCSK9* variant in the Japanese FH population. The five most frequent *LDLR* pathogenic variants, found in about 17% of this FH cohort, were identified: *LDLR* c.1845 + 2 T > C, c.1012T > A (p.C338S), c.1297G > C (p.D433H), c.1702C > G (p.L568V), and c.2431A > T (p.K811X). This study found that *LDLR* and *PCSK9* pathogenic variants were common in heterozygous Japanese FH patients (Hori et al., 2019).

*APOB* mutations, especially *APOB* c.10579 C > T (p.R3527W), were common mutations in Taiwan (9.2%) (Chiou and Charng, 2012) and China (10.9%) (Sun et al., 2018). However, it was not detected in Japan in the past (Nohara et al., 1995; Yu et al., 2002). The first Japanese case of *APOB* mutation, *APOB* c.10580 G > A (p.Arg3527Gln), was just reported recently (Hori et al., 2020). The origin of this *APOB* mutation was unknown and might be imported from other Asian countries. In contrast, *PCSK9* mutations, especially *PCSK9* c.94G > A (p.E32K), were quite prevalent in Japan (7.8%) (Hori et al., 2019), but have not been detected in Taiwan and were very rare in other Asian regions.

### Korea

FH genetic studies in Korea are summarized in Table 5.

**TABLE 5:** Genetic diagnosis of familial hypercholesterolemia in Korea.

**Case numbers**	**Age**	**Male**	**Diagnosis criteria**	**Genotyping**	**Main Findings**	**References**
28	NM	NM	(1) Plasma cholesterol levels > 300 mg/dl in adults and > 250 mg/dl in children, (2) a positive family history of hypercholesterolemia, Achilles tendon xanthomas, and coronary artery disease.	Southern blot hybridization with probes encompassing exons 1–18 of the *LDLR* gene	Two large deletion mutations of the *LDLR* gene were identified from three FH patients (10.7%).	Chae et al., 1997
80	NM	NM	(1) Plasma cholesterol levels > 300 mg/dl in adults and > 250 mg/dl in children, (2) a positive family history of hypercholesterolemia, Achilles tendon xanthomas, and coronary artery disease.	DNA sequencing for small structural rearrangements of *LDLR* gene	Three small deletions in exon 11 (FH 2, FH 34, and FH 400) were identified from three unrelated FH families.	Chae et al., 1999
45	NM	NM	LDL-C > 2.6 g/L, a positive family history of hypercholesterolemia or early coronary artery disease, and Achilles tendon xanthomas	Long-distance PCR for *LDLR* gene	Two large deletion mutations (FH6 and FH 32) were identified in four families	Kim et al., 1999
20	NM	NM	(1) Plasma cholesterol levels > 300 mg/dL in adults and > 250 mg/d: in children, (2) a positive history of hypercholesterolemia or early coronary artery disease, and Achilles tendon xanthomas.	DNA sequencing for *LDLR* gene by single-strand conformation	*LDLR* mutations were identified in 5 patients (25.0%).	Shin et al., 2000
489	NM	NM	244 members from 15 normal and 28 FH pedigrees 245 individuals, including 187 normal and 58 FH	Restriction fragment length polymorphisms	Among the 512 possible combinations for the nine polymorphic sites, there were 39 unique haplotypes detected. The four most common haplotypes accounted for 59.4% of those sampled. The haplotypes indicated marked linkage disequilibrium for these 10 sites and throughout the region containing the *LDLR* gene.	Chae et al., 2001
31	NM	NM	Total cholesterol >290 mg/dL (age > 40) or >220 mg/dL (age < 40), with tendon xanthoma and/or coronary heart disease, or with first-degree relatives who had hypercholesterolemia.	DNA sequencing for *LDLR* gene by single-strand conformation and by heteroduplex formation	Sixteen different mutations of the *LDLR* gene were identified in 25 unrelated Korean patients with HeFH (80.6%).	Kim et al., 2004
69	54	29 (42.0%)	Simon Broome criteria	Whole-exome sequencing	Genetic mutations were detected in 23 patients (33.3%).	Han et al., 2015
97	NM	NM	Unrelated patients older than 19 years who had (1) LDL-C > 190 mg/dL without lipid lowering agents and tendon xanthoma or (2) the same LDL-C levels and a family history of coronary artery disease or hypercholesterolemia	Whole exome sequencing, targeted exome sequencing, and Sanger sequencing	Putative pathogenic mutations in *LDLR*, *APOB*, or *PCSK9* genes were identified in 31 patients (32%).	Shin et al., 2015
97	54.1	38 (39.2%)	Unrelated patients older than 19 years who had (1) LDL-C > 190 mg/dL without lipid lowering agents and tendon xanthoma or (2) the same LDL-C levels and a family history of coronary artery disease or hypercholesterolemia	SNP	Mutation-negative FH patients had higher SNP scores than controls. SNP score analysis could be used for identification of polygenic cause of FH in Korean patients	Kwon et al., 2015
283	63.4	47 (16.6%)	Total cholesterol levels ≥ 290 mg/dL (7.5 mmol/L)	NGS	Seventeen different mutations in *LDLR*, *APOB*, and *PCSK9* genes were identified by NGS in 23 patients (8.1%). For the 110 subjects with a total cholesterol ≥ 310 mg/dL, 10 variants were identified in 10 patients (9.1%)	Kim et al., 2018

*APOB, apolipoprotein B; DNA, deoxyribonucleic acid; FH, familial hypercholesterolemia; LDL-C, low-density lipoprotein cholesterol; *LDLR*, low-density lipoprotein receptor; NGS, next generation sequencing; NM, not mention; PCR, polymerase chain reaction; *PCSK9*, proprotein convertase subtilisin/kexin type 9; SNP, single nucleotide polymorphism.*

In 28 clinically diagnosed HeFH patients, genetic analysis was performed for *LDLR* gene by Southern blot hybridization. Three of them (10.7%) were diagnosed with two large *LDLR* deletion mutations (FH29 and FH110) (Chae et al., 1997).

In 80 clinically diagnosed FH patients, genetic analysis was performed to detect small structural rearrangements of the *LDLR* gene, which were undetected by southern blot hybridization. Three novel small deletions were identified (FH 2, FH 34 and FH 400) (Chae et al., 1999).

In 45 unrelated HeFH patients, genetic analysis was performed to assess the *LDLR* gene mutations by long-distance PCR. Two different large deletion mutations were identified (FH6 and FH 32) (Kim et al., 1999).

In 20 unrelated FH patients, genetic analysis was performed for the *LDLR* gene by single-strand conformation polymorphism. *LDLR* mutations were detected in five patients (25%). There were four novel point mutations, including one non-sense mutation and three missense mutations (Shin et al., 2000).

To study the role of the *LDLR* gene in polygenic hypercholesterolemia, a study was conducted in 244 members of 43 different pedigrees (15 normal and 28 FH pedigrees) and 245 individuals (187 normal and 58 FH) for 9 different restriction fragment length polymorphisms (RFLPs) (*Taq*I, *Stu*I, *Hin*cII, *Bst*EII, *Ava*II, *Pvu*II, MspIA, MspIB, and *Nco*I) and a sequence variation at Arg450. Frequencies of these polymorphisms were similar between FH patients and controls. Among the 512 possible combinations for the nine polymorphic sites, 39 unique haplotypes were detected. The four most common haplotypes accounted for 59.4% of the total patients sampled. The study found that the haplotypes indicated marked linkage disequilibrium for these 10 sites and throughout the region containing the *LDLR* gene (Chae et al., 2001).

In 31 clinically diagnosed HeFH patients, genetic analysis was performed for the *LDLR* gene by single-strand conformation and heteroduplex formation. *LDLR* gene mutations were detected in 25 patients (80.6%). There were sixteen different *LDLR* mutations. The study identified one mutation that had only been reported in a Korean FH patient, the in-frame 36-bp deletion (1591del36) in exon 11. Five novel mutations were identified, including *LDLR* c.311G > A (C104Y), c.661del17, c.1705insCTAG, c.2088C > A (C696X), and c.941-1G > A (Kim et al., 2004).

In 69 clinically diagnosed FH cases, whole-exome sequencing analysis was applied to identify genetic mutations in the 3 known FH-related genes (*LDLR*, *APOB*, and *PCSK9* genes). Genetic mutations were detected in 23 patients (33.3%). The most common genetic mutation was in the *LDLR* gene, which was identified in 19 patients (82.6%), included 2 copy number deletions and 17 mutations. There was also one *APOB* gene mutation and one *PCSK9* gene mutation. Two frameshift deletions in the *LDLR* gene and one *PCSK9* mutation were novel causative mutations. By co-segregation in their relatives, one copy number deletion and novel mutation were validated (Han et al., 2015).

One study enrolled patients with >190 mg/dL LDL-C and xanthoma or a family history of CAD or hypercholesterolemia. Among 97 patients, putative pathogenic mutations in *LDLR*, *APOB*, or *PCSK9* genes were identified in 31 patients (32%). When comparing the four clinical diagnosis criteria of FH (Simon Broome, Dutch, MEDPED, and Japanese), mutation detection rates were higher using the MEDPED criteria (67–75%) and lower in the Simon Broome or Dutch criteria (35%–37%). The study found that 225 mg/dL was the best LDL-C threshold for putative mutations in Korean FH patients (Shin et al., 2015).

The polygenic cause of FH in Korean patients was investigated in 97 FH patients and 2,274 controls from the Korean Health Examinee (HEXA) shared control study. These patients received genotyping for 12 single nucleotide polymorphisms (SNPs) used in prior studies to assess the polygenic causes of FH in Caucasian patients and 4 SNPs associated with LDL-C levels in East Asians. The study found that mutation-negative FH patients had higher SNP scores than controls. The study also showed that SNP score analysis could be used for the identification of the polygenic cause of FH in Korean patients (Kwon et al., 2015).

A total of 283 subjects with total cholesterol levels ≥ 290 mg/dL (7.5 mmol/L) were selected from the Namwon and Dong-gu Studies. Seventeen different mutations in *LDLR*, *APOB*, and *PCSK9* genes were identified by NGS in 23 patients (8.1%). For the 110 subjects with a total cholesterol ≥ 310 mg/dL, 10 variants were identified in 10 patients (9.1%). There were two novel *LDLR* variants, including *LDLR* c.2038 (p.L680V), and c.2201 (p.T734F) (Kim et al., 2018).

### Malaysia

FH genetic studies in Malaysia are summarized in Table 6.

**TABLE 6:** Genetic diagnosis of familial hypercholesterolemia in Malaysia.

**Case numbers**	**Age**	**Male**	**Diagnosis criteria**	**Genotyping**	**Main Findings**	**References**
154	44.6	73 (47.4%)	Simon Broome criteria	The promoter region and exons 2–15 of the *LDLR* and MLPA for large rearrangement	Among these patients, 117 subjects (76.0%) had total 29 *LDLR* variants.	Al-Khateeb et al., 2011
140	47.0	73 (52.1%)	Dutch Lipid Clinic Network criteria	Selected SNPs of *LDLR*, *APOB*, *PCSK9*, and other genes	Significant differences in allele frequency among Malaysians and other populations (European Whites, Han Chinese, Yoruba and Gujarati Indians) were noted in 23 markers when comparing to publicly available data.	Alex et al., 2012
141	46.8	73 (51.8%)	Dutch Lipid Clinic Network criteria	A total of 1,536 SNPs by using high throughput microarray genotyping platform	Amongst the FH cases, 108 out of 141 (76.60%) have had at least one significant risk-associated SNP.	Lye et al., 2013

*APOB, apolipoprotein B; *LDLR*, low-density lipoprotein receptor; *PCSK9*, proprotein convertase subtilisin/kexin type 9; MLPA, Multiplex ligation dependent probe analysis; SNP, single nucleotide polymorphism.*

A total of 154 unrelated FH patients from Malaysia (Kelantan) were analyzed for *LDLR* gene mutations. Among these patients, 117 subjects (76.0%) had a total of 29 *LDLR* variants. The most frequent variant was *LDLR* c.1060 + 7 T > C (*n* = 18, 11.7%). The second most frequent variant was c.1411A > G (p.R471G) (*n* = 17, 11.0%) in *LDLR*. Eight of them [*LDLR* c.300 C > T (p.D100D), c.415G > C (p.D139H), c.1411A > G (p.R471G), c.1705 + 117 T > G, c.1186 + 41T > A, 1705 + 112C > G, Dup exon 12, and c.1966_2010del17 (p.W666Fs)] were reported for the first time. The study also found that patients with pathogenic mutations had higher LDL-C levels and a higher incidence of tendon xanthoma and cardiovascular diseases than those with non-pathogenic variants (Al-Khateeb et al., 2011).

A total of 140 clinically diagnosed autosomal dominant hypercholesterolemia (ADH) and 111 healthy control subjects were analyzed for selected SNPs in *LDLR*, *APOB*, *PCSK9*, and other genes. The ADH patients included Malays (60.0%), Indians (25.0%), and Chinese (15.0%). A total of 310 markers were examined, including 73 from *LDLR*, 130 from *APOB*, and 107 from *PCSK9*. Significant differences in allele frequency among Malaysians and other populations (European Whites, Han Chinese, Yoruba, and Gujarati Indians) were noted in 23 markers when compared to publicly available data. These markers included 2 on *LDLR*, 17 on *APOB*, and 4 on *PCSK9* (Alex et al., 2012).

In another study, 141 patients with clinically diagnosed FH and 111 unrelated control subjects were genetically analyzed via a high throughput microarray genotyping platform. Among the FH cases, 108 (76.60%) had at least one SNP that was associated with FH risk. Eleven of the 14 SNPs were significantly associated with an increased risk of FH, including 1 SNP in *LDLR*, 7 in *APOB*, and 3 in *PCSK9*. The study showed that *APOB* rs12720762 was associated with the highest risk of FH (odds ratio 14.78, *p* < 0.001) (Lye et al., 2013).

Two independent studies from the same team in Malaysia used SNPs for genetic analysis (Alex et al., 2012; Lye et al., 2013). Although they found SNPs with different allele frequencies between Malaysians, European Whites, Han Chinese, Yoruba, and Gujarati Indians, the functional significance of these SNPs requires further investigation.

### Singapore

FH genetic studies in Singapore are summarized in Table 7.

**TABLE 7:** Genetic diagnosis of familial hypercholesterolemia in Singapore.

**Case numbers**	**Age**	**Male**	**Diagnosis criteria**	**Genotyping**	**Main Findings**	**References**
96	33	77 (80.2%)	Simon Broome criteria or LDL-C > 4.9 mmol/L with unknown family history	NGS in 26 lipid-related genes	Among them, 50 patients (52.1%) had *LDLR* mutations and 4 patients (4.2%) had *APOB* mutations.	Pek et al., 2018

*APOB, apolipoprotein B; LDL-C, low-density lipoprotein cholesterol; *LDLR*, low-density lipoprotein receptor; NGS, next generation sequencing.*

In Singapore, 96 clinically suspected FH patients were genetically analyzed via NGS of 26 lipid-related genes. The cohort included Chinese (78.1%), Malays (13.5%), Indians (5.2%), and other ethnicities. A total of 50 patients (52.1%) had *LDLR* mutations and 4 (4.2%) had *APOB* mutations. No *PCSK9* mutations were detected in this cohort. There were 15 novel *LDLR* mutations. The study concluded that the mutation distribution was similar to other Asian countries, but the spectrum was different locally (Pek et al., 2018).

### Philippines

FH genetic studies in Philippines are summarized in Table 8.

**TABLE 8:** Genetic diagnosis of familial hypercholesterolemia in Philippines.

**Case numbers**	**Age**	**Male**	**Diagnosis criteria**	**Genotyping**	**Main Findings**	**References**
60	55	21 (35.0%)	Dutch Lipid Clinic Network criteria	*LDLR* mutation identified by denaturing high performance liquid chromatography and DNA sequencing	Twelve patients (20%) had documented *LDLR* mutations and six of the mutations were considered novel.	Punzalan et al., 2005

*DNA, deoxyribonucleic acid; *LDLR*, low-density lipoprotein receptor.*

In the Philippines, 60 suspected FH patients were analyzed for *LDLR* mutations. *LDLR* mutations were detected in 12 patients (20%), and six novel mutations were identified. The study found that *LDLR* mutations were significantly associated with LDL-C levels, FH scores, and a family history of dyslipidemia (Punzalan et al., 2005).

In addition to *LDLR* mutations, further studies are still needed to identify relevant mutations in *APOB*, *PCSK9*, and other FH-related genes in this population.

### Sri Lanka

FH genetic studies in Sri Lanka are summarized in Table 9.

**TABLE 9:** Genetic diagnosis of familial hypercholesterolemia in Sri Lankan.

**Case numbers**	**Age**	**Male**	**Diagnosis criteria**	**Genotyping**	**Main Findings**	**References**
27	50 (24–73)	13 (48.1%)	Modified Simon Broome criteria or Dutch Lipid Clinic Network criteria	Sanger sequencing	Pathogenic missense mutations were detected only in 5 patients (18.5%).	Paththinige et al., 2018

In Sri Lanka, a total of 27 clinically diagnosed FH patients were analyzed for *LDLR* mutations using Sanger sequencing. Among these patients, pathogenic missense mutations were detected in only five (18.5%). Four patients were heterozygous for *LDLR* mutations, including *LDLR* c.682 G > C (p.E228Q) in 2 patients, *LDLR* c.1720C > A (p.R574S) in 1, and *LDLR* c.1855 T > A (p.F619L) in 1. One patient was compound heterozygous for *LDLR* c.2289 G > T (p.E763D) and *LDLR* c.1670 C > G (p.T557S). The study found that *LDLR* mutations were markedly low in this population (Paththinige et al., 2018).

This is the first report on *LDLR* mutations in this population. However, the detection rate of genetic mutations was low. Furthermore, the study did not screen for other FH mutations, including those in *APOB*, *PCSK9*, and *LDLRAP1*.

### India

FH genetic studies in India are summarized in Table 10.

**TABLE 10:** Genetic diagnosis of familial hypercholesterolemia in India.

**Case numbers**	**Age**	**Male**	**Diagnosis criteria**	**Genotyping**	**Main Findings**	**References**
100	39 (3–74)	63 (63.0%)	Dutch Lipid Clinic Network criteria (adapted for the Indian population)	Sanger sequencing, MLPA, and NGS	Genetic mutations were identified in 47 cases (47.0%). Based on modified Dutch Lipid Clinic Network criteria, mutations were detected in 91.4% of definite FH cases, in 40% of probable FH cases, and in 18.8% of possible FH cases.	Setia et al., 2020

*FH, familial hypercholesterolemia; MLPA, Multiplex ligation dependent probe analysis; NGS, next generation sequencing.*

In one Indian study, a total of 100 potential FH cases were genetically analyzed using Sanger sequencing, MLPA, and NGS. Genetic mutations were identified in 47 cases (47.0%). Based on modified Dutch Lipid Clinic Network criteria, mutations were detected in 91.4% of definite FH cases, in 40% of probable FH cases, and in 18.8% of possible FH cases. A total of 38 pathogenic variants were identified, including 33 *LDLR* mutations, 3 *APOB* mutations, and 2 *PCSK9* mutations. There were ten novel pathogenic variants. Interestingly, a likely founder mutation in intron 10 (c.1587-1 G > A) of the *LDLR* gene was observed in 6 North Indian families. However, the conventional pathogenic variants in previously reported *LDLR*, *APOB*, and *PCSK9* mutations were not detected. This study found different genetic variants between the Indian population and Western populations (Setia et al., 2020).

In addition, there were other genetic studies as abstracts from India. However, we did not report them in this review article.

## Genetic Differences of FH Between Different Ethnicities

Among the genetic studies in Asia, the detection rates of genetic mutations varied. The differences may be due to either the diagnostic methods used or the ethnicities sampled. With the advances in genetic diagnostic methods, detection rates will be further improved. Most of the genetic studies in Asia were confined to one ethnicity. Only a few studies enrolled FH patients with different ethnicities. Data from Singapore revealed a similar distribution of mutations as compared to other Asian countries (Pek et al., 2018). Data from Malaysia revealed significant differences in the allele frequency of markers for *LDLR*, *APOB*, and *PCSK9* genes compared to publicly available data from other populations (Alex et al., 2012). Until now, there is limited literature discussing the genetic differences in FH between Asian and non-Asian populations. Further studies are still needed to compare the genetic differences in FH between different ethnicities.

## Conclusion

Although there has been a continuous improvement in FH detection rates, there are still important gaps to fill in Asian countries in comparison to other countries and the United Kingdom. FH awareness, knowledge, and perception remain low in Asian physicians, particularly in less economically developed countries. More efforts are required to close this gap, such as extensive education and training programs. Although there have been some FH genetic studies in the Asian population, each utilizing different techniques, further studies are required to understand the different genetic backgrounds of FH in Asia.

## Author Contributions

C-CH and M-JC contributed to conception and design of the study, manuscript revision, read, and approved the submitted version. C-CH wrote the first draft of the manuscript.

## Conflict of Interest

The authors declare that the research was conducted in the absence of any commercial or financial relationships that could be construed as a potential conflict of interest.
